# Delivery and Biosafety of Oncolytic Virotherapy

**DOI:** 10.3389/fonc.2020.00475

**Published:** 2020-04-16

**Authors:** Lizhi Li, Shixin Liu, Duoduo Han, Bin Tang, Jian Ma

**Affiliations:** ^1^Hunan Cancer Hospital and the Affiliated Cancer Hospital of Xiangya School of Medicine, Central South University, Changsha, China; ^2^Cancer Research Institute, School of Basic Medical Science, Xiangya School of Medicine, Central South University, Changsha, China; ^3^Hunan Key Laboratory of Nonresolving Inflammation and Cancer, National Health Commission Key Laboratory of Carcinogenesis, Key Laboratory of Carcinogenesis and Cancer Invasion of Ministry of Education, Changsha, China

**Keywords:** oncolytic virotherapy, tumor, immunotherapy, delivery route, biosafety

## Abstract

In recent years, oncolytic virotherapy has emerged as a promising anticancer therapy. Oncolytic viruses destroy cancer cells, without damaging normal tissues, through virus self-replication and antitumor immunity responses, showing great potential for cancer treatment. However, the clinical guidelines for administering oncolytic virotherapy remain unclear. Delivery routes for oncolytic virotherapy to patients vary in existing studies, depending on the tumor sites and the objective of studies. Moreover, the biosafety of oncolytic virotherapy, including mainly uncontrolled adverse events and long-term complications, remains a serious concern that needs to be accurately measured. This review provides a comprehensive and detailed overview of the delivery and biosafety of oncolytic virotherapy.

Oncolytic virotherapy has been recognized as a promising new treatment for cancer in recent years. Oncolytic viruses are genetically modified or naturally occurring viruses that selectively replicate in cancer cells and kill them without damaging normal cells (1). The idea of using viruses to treat cancer patients was originated in the 1950s (2). Many cancer patients were treated with oncolytic virus preparations administered by almost every feasible route, and some of them had tumor regression over different time scales (3). In a study from Osaka University, tumor regressions were reported in 37 of 90 terminal cancer patients who received non-attenuated mumps virus treatment (4). Since then, researchers have increasingly focused on oncolytic viruses for cancer treatment. The mechanism of oncolytic virotherapy includes two main aspects: (1) after infection, oncolytic viruses inhibit protein synthesis of cancer cells and destroy infected cancer cells by virus self-replication, and (2) oncolytic viruses recruit and activate tumor-infiltrating immune cells by promoting the release of a large amount of tumor antigens and cytokines, thus inducing strong antitumor immunity responses (5–7). As a new cancer therapy strategy, oncolytic virotherapy has immeasurable application potential, bringing new hope to cancer patients. This review summarizes the delivery of oncolytic viruses to patients based on a scan of existing preclinical and clinical studies, including those on intratumoral, intravenous, intraperitoneal, limb perfusion, aerosol delivery, etc. (8). Currently, the commonly used oncolytic viruses include herpes simplex virus type 1 (HSV-1), oncolytic adenovirus, oncolytic pox virus, Newcastle disease virus, and reovirus. A large number of natural and genetically modified oncolytic viruses have been developed and have reached the clinical research stages (9). However, biosafety issues remain a matter of serious concern. The primary problem in oncolytic virotherapy is the risk of uncontrolled replication *in vivo* and possible transmission to patients' contacts, such as other patients and health care workers (10). In recent years, clinical trials to address these concerns have been conducted. In this review, the route of delivery and the biosafety of oncolytic virus are discussed. All oncolytic viruses included in this review are summarized in Supplementary Table 1.

## Oncolytic Virotherapy Emerged as a New Weapon Against Cancer

To date, three oncolytic virus drugs have been approved for cancer therapy. Rigvir (Riga virus) is an unmodified Echo virus that became the first approved oncolytic virus in the world for the treatment of melanoma in 2004 (11); Oncorine is an attenuated adenovirus that became the first clinically approved oncolytic virus in China in 2005 and the first approved recombinant oncolytic virus in the world for the treatment of head and neck tumors combined with chemotherapy (12). T-VEC, a recombinant human HSV-1, was approved by the U.S. Food and Drug Administration (FDA) in 2015 for the treatment of unresectable metastatic melanoma and was subsequently approved in the European Union for the treatment of locally advanced or metastatic cutaneous melanoma (13). The efficacy of oncolytic viruses on other types of tumors, such as lung cancer, liver cancer, pancreatic cancer, ovarian cancer, breast cancer, prostate cancer, bladder cancer, glioma, etc., is currently being addressed in clinical research and remains largely unknown (9). Recent clinical studies have shown the benefit of oncolytic virotherapy on some refractory malignant tumors, such as glioblastoma and triple-negative breast cancer (14–16).

Oncolytic viruses can also be used for tumor imaging with molecular imaging techniques. An oncolytic virus carrying a reporter gene can selectively replicate and express the reporter gene in the tumor cells such that the tumor cells emit fluorescence and absorb exogenous radionuclides. The tumors can be accurately imaged by bioluminescent detection systems such as CT (17). Human sodium iodide synergistic transporter protein (hNIS) was combined with human somatostatin receptor 2 (hSSR2) to engineer oncolytic viruses. After systemic administration of this virus, radioisotopes (^99^Tc and ^131^I) were administered, resulting in accumulation of the isotopes in the tumor mass, thereby enabling the tumor to be observed and located in a mouse model using a SPECT/CT imaging system (18). A combination of an exogenous lysine-rich protein (LRP) gene with the HSV genome can be used to image tumors by MRI because this construct changes the magnetic field associated with the metabolism rate of the tumors (19). The accurate imaging of tumors by oncolytic viruses has shown broad application prospects for early diagnosis and localization and visualization of tumors (18, 19).

**Figure 1:**
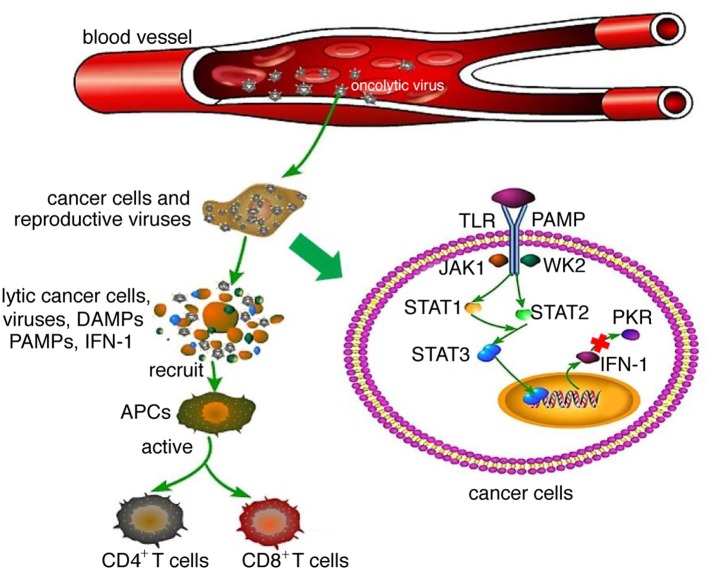
Mechanism of oncolytic virotherapy. When oncolytic viruses attack a normal cell, viruses activate JAK-STAT or NF-κB pathways through interaction between TLRs and PAMPs, which induce type I IFN transcription and release. Then, type I IFN activates PKR, which is essential for regulating abnormal cell proliferation and innate cellular antiviral responses. However, when oncolytic viruses attack cancer cells, interferon signaling and PKR activity are inhibited; thus, virus clearance is blocked, enabling virus replication. Following virus replication, most oncolytic viruses can induce cell death, at which time they release not only tumor-associated antigens that can promote an adaptive immune response but also viral PAMPs and additional cellular DAMPs and cytokines. These released molecules recruit antigen-presenting cells (APCs) and promote their maturation, subsequently activating antigen-specific CD4^+^ and CD8^+^ T cell responses, enabling CD8^+^ T cells to expand into cytotoxic effector cells and mediate antitumor immunity.

Oncolytic viruses are thought to mediate antitumor activity through two different mechanisms: selective replication within tumor cells, which results in a direct lytic effect on the tumor cells, and induction of a systemic antitumor immunity response. After an oncolytic virus infects normal cells, it activates intracellular Toll-like receptors (TLRs) through pathogen-associated molecular patterns (PAMPs, including elements of viral capsids, DNAs, RNAs and protein products), thus activating the JAK–STAT or NF-κB pathway, inducing type I interferon (IFN) transcription and release (6). The interferon-induced double-stranded RNA-dependent protein kinase (PKR) can be activated by type I interferon and TLR and is essential for regulating cell proliferation and innate cellular antiviral responses. The activation of PKR inhibits cellular protein synthesis, which subsequently blocks cell proliferation and inhibits viral propagation (20). In cancer cells, interferon signaling and PKR activity are inhibited; thus, virus clearance is blocked, enabling virus replication (6, 20) (Figure 1). Following virus replication, most oncolytic viruses induce cell death, triggering not only the release of tumor-associated antigens that can promote an adaptive immune response but also viral PAMPs, cellular danger-associated molecular pattern signals (DAMPs; for example, heat shock proteins, HMGB1 protein, calreticulin, ATP and uric acid), and cytokines (for example, type I interferon, TNFα and IL-12). These released molecules recruit antigen-presenting cells (APCs) and promote their maturation, subsequently activating antigen-specific CD4^+^ and CD8^+^ T cell responses, enabling CD8^+^ T cells to expand into cytotoxic effector cells and mediating antitumor immunity (6, 15). The local release of interferons, chemokines and DAMP and PAMP factors activates tumor-infiltrating immune cells, which contribute to the reversal of the immune suppressive state of the tumor microenvironment and promote effective antitumor responses (5, 7, 15).

Since the antitumor activity of an oncolytic virus is not enough to effectively eliminate tumors, various strategies have been designed to improve their efficacy. The main strategies have been genetic modification, combined treatment and increasing the extent of virus replication and transmission.

### Genetical Modification of Oncolytic Viruses

Genetic manipulation of the viral genome to create non-pathogenic viruses has become the main technique of oncolytic virus development with the goals of weakening virus pathogenicity, enhancing target selectivity, reducing adverse reactions, and/or inserting exogenous therapeutic genes into the virus genome, thereby increasing their expression in tumors and enhancing the treatment effect of the oncolytic virus (5). Deletion of ICP6 in the HSV-1 genome can weaken the pathogenicity of the virus; deletion of the gamma 34.5 gene can reduce the neurotoxicity of HSV-1 and enhance its selective replication in tumor cells. Introduction of microRNA targeting sites in the HSV-1 genome can inhibit viral gene expression and translation into normal cells that express specific microRNAs and improve the tumor cell selectivity of the virus (21). The oncolytic virus expressing PGE2-inactivating enzyme HPGD after genetic modification can reduce the level of myeloid-derived suppressor cells (MDSCs), thereby breaking down the immunosuppressive microenvironment of tumors and enhancing the sensitivity of oncolytic virotherapy (22). A genetically modified oncolytic adenovirus rich in CpG sites can overstimulate TLR9, thereby activating innate and adaptive immune responses and enhancing antitumor activity (23). Introducing oncogenesis-related gene-specific siRNA into an adenovirus genome can suppress the expression of oncogenes and inhibit tumor growth. Construction of adenoviruses expressing specific cytokines (such as GM-SF, IL-2, IL-12, etc.) can induce an antitumor immune response and enhance the oncolytic effects of the virus (24).

### Combination Therapies With Oncolytic Viruses

An attractive feature of an oncolytic virus is that it can be combined with other immunotherapy approaches, among which immunological checkpoint inhibitor-combined therapy has become a mainstream strategy. The elevated expression of PD-L1 in the tumor microenvironment inhibits the infiltration of immune cells, which results in an immunosuppressed tumor microenvironment, which restricts the antitumor effect of oncolytic viruses. The combined approach of an oncolytic virus and a PD-1 or PD-L1 blockade can enhance antitumor immunity and the oncolytic effect (25–28). Additionally, the combination of oncolytic virotherapy and CAR-T immunotherapy produces a synergistic effect in cancer patients. Oncolytic viruses can induce tumor cell lysis and the release of tumor-associated antigens, thus stimulating the immune response to tumors and overcoming the obstacles associated with CAR-T applied to solid tumors. At the same time, the CAR-T antitumor effect on metastatic tumor sites overcomes the limitations of the oncolytic virus. The combination of these two approaches enhances the antitumor activity and has great application prospects (29, 30). Oncolytic viruses combined with chemotherapy have shown a promising effect. It is possible to combine oncolytic viruses with different immune characteristics to overcome antiviral immune responses and exert synergistic effects. Similarly, it has been demonstrated that bacteria can synergize with oncolytic viruses (31). For example, Le Boeuf et al. demonstrated that VSV (Vesicular Stomatitis Virus) combined with VACV (Vacienia Virus) improved antitumor response in immunodeficient and immunocompetent mouse tumor models (32). Cronin et al. showed that intravenous application of nonpathogenic *E. coli* expressing the vaccinia type 1 IFN antagonist B18R augmented subsequent therapy with oncolytic VSV by overcoming innate immunity against oncolytic viruses in an athymic nude mouse model (33).

### Improvement in the Replication and Transmission of Oncolytic Viruses

Multiple strategies are used to overcome the obstacle of immune clearance, which challenges oncolytic virus therapy. Using stem cells as oncolytic virus carriers can reduce the immunogenicity of the viruses, while modifying the surface of oncolytic viruses with polymers and liposomes can increase the transmission of the viruses and enhance their antitumor effect (34, 35). The extracellular matrix (ECM), as a physical barrier, interferes with the transmission of oncolytic virus in a solid tumor mass, therefore, reconfiguration of the ECM can enhance the transmission of a virus into tumors. For instance, relaxin can inhibit the expression of collagen and the formation of ECM, and a decolorant can change the structure of ECM-residing collagen to remodel the ECM (24). Oncolytic adenoviruses expressing relaxin, which selectively degrades aberrant ECM, generated a potent antitumor effect through the effective induction of apoptosis (36). Most viruses can be engineered to encode exogenous genes. Expression of the transcription inhibitor PRd1-bf1/blimp1 induced by vascular endothelial growth factor (VEGF) can decrease the expression of type I interferon, thus weakening the antiviral immunity of vascular endothelial cells and promoting virus replication and transmission in tumors (37). Expression of interferon antagonists by gene modification can reduce the expression level of interferon, which weakens antiviral immunity, thereby promoting virus replication and enhancing the antitumor activity and transmission of oncolytic viruses (38).

## Delivery Routes of Oncolytic Viruses

**Figure 2:**
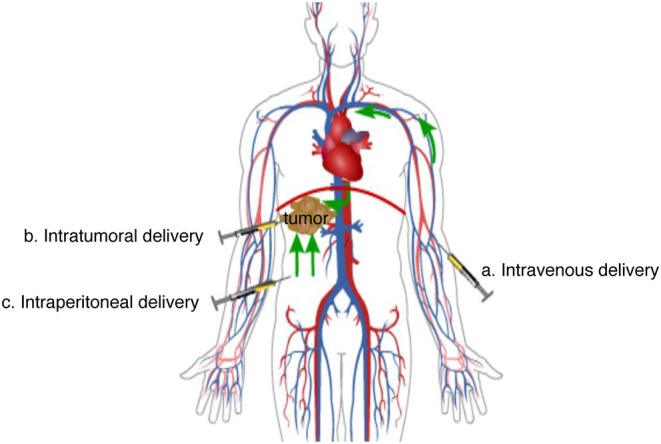
Main delivery routes of oncolytic virotherapy. (a) Intravenous delivery: When oncolytic viruses are injected into the peripheral vein, they reach tumor lesions in non-specific organs and systems through the circulation system. (b) Intratumoral delivery: When oncolytic viruses are injected into tumors, they have a direct therapeutic effect on the lesion. (c) Intraperitoneal delivery: When oncolytic viruses are injected into the peritoneal cavity, they will be absorbed into the veins of the peritoneum and then reach tumor lesions of some organs or systems through the circulation system, or they will diffuse directly to tumor lesions in the peritoneal cavity.

Suboptimal delivery is a potential cause of treatment failure. Multiple routes of delivery have been investigated for oncolytic virotherapy (Figure 2), the selection of which is critical for therapy efficiency. Researchers choose different routes of delivery according to their research objectives and the available and necessary experimental materials.

### Direct Intratumoral Delivery

Intratumoral delivery is the most common route of oncolytic virus delivery. The concentration of oncolytic virus in the target site can be accurately controlled, and at the same time, the side effects caused by the virus being mistargeted to other organs can be prevented. Intratumoral delivery is much more suitable for surface tumors such as melanoma than it is for deep tumors such as glioblastoma due to operational difficulties in delivery.

Maintaining the concentration of oncolytic virus in the target site is a crucial advantage of intratumoral delivery, and researchers tend to observe more definite therapeutic effects with this method (39–41). Intratumoral delivery enables researchers to control the precise concentration of the oncolytic virus at the tumor site and to compare the results of *in vitro* experiments with those of *in vivo* studies (26, 42, 43). However, the risks and expenses associated with the complex procedures involved in intratumoral delivery make repeat dosing *in vivo* difficult.

At the level of cellular and animal models, Streby et al. demonstrated that HSV1716 (an oncolytic herpesvirus) was safely applied by direct intratumoral delivery (44). Dimethyl fumarate and various other fumaric acid and maleate (FMAE) compounds can enhance the ability of an oncolytic virus to infect melanoma cancer cells by direct intratumoral delivery (39). Warner et al. demonstrated that early expression of hNIS in colon cancer cells made viral replication reliably imageable via positron emission tomography (PET) of I-124 uptake (45). Direct intratumoral injection of oncolytic adenovirus VCN-01 for the treatment of retinoblastoma has also been shown to be effective (46). Choi et al. proved that CF33 (a novel chimeric orthopoxvirus encoding luciferase, enabling real-time view of cell infection), was effective *in vitro* with potent cytotoxicity and efficient intracellular replication observed in triple-negative breast cancer (TNBC, an aggressive subtype of breast cancer with high recurrence rate and poor prognosis) lines with PI3K/AKT pathway mutations (47). Jung et al. constructed two mathematical models to analyze the spread and expression of oncolytic measles viruses administered by direct intratumoral injection *in vivo* (42).

At the preclinical level, Bartee et al. demonstrated that tumors can secrete a soluble form of programmed cell death protein 1 (PD1) upon intratumoral injection of a novel recombinant myxoma virus (vPD1), thus enhancing the effect of the oncolytic virotherapy (26). Kicielinski et al. reported a multicenter study which demonstrated the approach of delivering intratumoral infusion of reovirus to patients with recurrent malignant glioma to be safe and well-tolerated (48). Intratumorally injected oncolytic adenovirus that had been engineered to encode a bispecific antibody (T-cell-targeted substances), combined with direct virolysis, induced endogenous T-cell activation to attack stromal fibroblasts, providing a multimodal treatment strategy within a single therapeutic agent (43). Blockade of immune checkpoints, immunogenic chemotherapy and IFN-α suppression can promote the local therapeutic effect of oncolytic viruses by direct intratumoral injection (41). Antagonizing the glycolysis and carboxylation of glutamine can enhance the activity of an oncolytic adenovirus directly injected into tumors by promoting its lysis in the cancer cells (49).

At the clinical trial level, Hirooka et al. conducted a phase I clinical trial by intratumoral injection. It was shown that HF10 injection of oncolytic virus was effective for unresectable locally advanced pancreatic cancer (50). On this basis, Nakao et al. conducted a phase I clinical trial of oncolytic virus HF10 with increasing doses for pancreatic cancer (51). Intratumoral injection of reovirus is effective in patients with recurrent malignant glioma, which has been histologically confirmed in a phase I clinical trial (52).

### Intravenous Delivery

Many researchers prefer using intravenous injection to intratumoral injection in the clinical trial stage, which may be related to the complexity of the operation as well as the hurdles for distant metastasis of intratumoral delivery. Intravenous delivery of oncolytic virus represents a more simplified administration route for physicians.

For central nervous system tumors, Samson et al. demonstrated that oncolytic reovirus (ORV) vaccination by intravenous administration can block subsequent immune checkpoints in patients with brain tumors (53). The combination of oncolytic virus with CXCR4 antagonism can enhance the antitumor effect of dendritic cells in the context of neuroblastoma by intravenous administration (54). Tang et al. explored the selectivity of the oncolytic virus poxvirus to central nervous system tumors by intravenous administration (55). The therapeutic effect of oncolytic virus HSV G207 is obvious for children with progressive or recurrent malignant supratentorial brain tumors in a phase I clinical trial by means of intravenous delivery (56). These clinical trials confirmed that some oncolytic viruses can reach brain tumor tissues by bypassing the blood-brain barrier.

In addition to central nervous system tumors, intravenous administration has been applied to tumors in other organs and systems of the body. Saito et al. demonstrated that intravenous delivery of oncolytic adenovirus-carrying tumor vaccine into mouse squamous cell carcinoma models can inhibit the growth of multiple lung tumors (57). Oncolytic virus M1 was used to treat invasive bladder cancer by intravenous administration (58). Intravenous administration of oncolytic virus can modify the tumor microenvironment of prostate cancer and thus inhibit the growth of prostate cancer (59). Paclitaxel combined with oncolytic reovirus is effective in the treatment of recurrent ovarian cancer, fallopian tube cancer and peritoneal cancer through intravenous administration in a phase III clinical trial (60). Moreover, Nguyen et al. evaluated the polymer shielding effect of oncolytic adenovirus (Ad6) used to treat human prostate cancer by intravenous administration (61). Huang et al. explored the possibility of editing oncolytic virus vaccines using functional peptides by intravenous administration (62). Intravenous administration of oncolytic virus T-VEC combined with ipilimumab was successfully used to treat stage IIIb-IV melanoma, which could not be treated or resected in the past (63). The effectiveness of intravenous injection of oncolytic measles virus was also observed in the treatment of atypical teratoid rhabdomyoma in a xenotransplantation mouse model (64).

### Other Routes of Delivery

In addition to the above two main methods, researchers also apply other routes of delivery. Chen et al. conducted an experiment using intraperitoneal injection of oHSV-1 to mice, suggesting that the combination of a PD-1 blockade and oHSV-1 may be an effective treatment strategy for childhood soft-tissue sarcoma (65). Besides, low dose of CF33 was confirmed to treat pancreatic cancer by intraperitoneal injection *in vivo* experiments (66). Kuryk et al. mainly used subcutaneous administration to indicate the clinical safety of application for Phase I clinical studies of ONCOS-102 (Ad5/3-D24-GM-CSF) for therapy for advanced cancers (67). Ochiai et al. administered PVS-RIPO into the spinal cord of transgenic mice, suggesting that intrathecal treatment with PVS-RIPO may be useful for the treatment of neoplastic meningitis in patients with glioblastoma multiforme (68).

Due to the large peritoneal area, intraperitoneal injected drugs can be absorbed faster than drugs administered the rough subcutaneous injection but slower than those delivered by intravenous injection. Because it is relatively easy to administer, intraperitoneal injection requires few specialty skills. Intraperitoneal injection is an ideal choice for targeting the organs in the abdominal cavity. Subcutaneous injection is the common method for administrating oncolytic viruses, but it is applied only to small animals in which veins are difficult to find. In addition, the scope of intrathecal injection is limited to central nervous system tumors. In other words, these delivery routes are used less frequently and are mainly limited to animal experiments mostly because of their low efficiency and narrow range of effectiveness.

Table 1 summarizes the different administration methods currently used for different types of tumors. The scientific community has not yet established a clear rubric to determine the advantages and disadvantages of using different delivery routes, which means that the best criteria for choosing the routes of delivery are debated.

**Table 1:** Delivery routes of oncolytic viruses in multiple tumors.

**Intratumoral administration**	**Intravenous administration**	**Intraperitoneal administration**	**Intrathecal administration**	**Subcutaneous administration**
Melanoma (39)	Melanoma (63)	Angiosarcoma (65)	Glioblastoma (63)	Melanoma (65)
Retinoblastoma (46)	Bladder cancer (58)	Epithelioid sarcoma (65)	Glioma (60)	Soft tissue sarcoma (65)
Pancreatic carcinoma (50)	Lung squamous cell carcinoma (57)	Kaposi's sarcoma (65)	Ependymoma (60)	
Astrocytoma (52)	Astrocytoma (53)	Gastrointestinal stromal tumor (65)	Primitive neuroectodermal tumor (60)	
Gliomas (48)	Neuroblastoma (54, 55)	Leiomyosarcoma (65)	Central nervous system lymphoma (60)	
Breast cancer (47)	Carcinoma ovarii (60)	Liposarcoma (65)		
Colorectal cancer (69)	Carcinoma of fallopian tube (60)	Pancreatic carcinoma (66)		
	Peritoneal carcinoma (60)			
	Prostatic carcinoma (59)			
	Atypical teratogenic rhabdoid tumor (64)			
	Glioblastoma (40)			

As shown in Table 2, there are advantages and disadvantages to the five routes of delivery. Intratumoral delivery can maintain the concentration of the oncolytic virus in the target site, and researchers tend to observe more definitive therapeutic effects (39–41). Furthermore, the application of intratumoral injection enables researchers to control the precise concentration of oncolytic virus in the tumor site and to compare the results of *in vitro* experiments with those of *in vivo* studies (26, 42, 43). However, the risks and expense associated with the complex procedures involved in intratumoral delivery make repeat dosing difficult.

**Table 2:** Advantages and disadvantages of two different routes for delivering oncolytic viruses.

	**Intratumoral delivery**	**Intravenous delivery**	**Intraperitoneal administration**	**Intrathecal administration**	**Subcutaneous administration**
Advantages	High concentration of oncolytic virus at target tissue to observe a definite effect (39–41)	Good choice when injecting oncolytic virus directly into tumors is challenging (26, 42, 43, 54, 55, 58, 59)	Absorbed faster than subcutaneous injection (65)	An ideal choice for CNS tumors (68)	Easy to operate (65)
	Enables researchers to control the precise concentration of the oncolytic virus in tumor sites (26, 42, 43)	Convenient and rapid for researchers at the clinical experimental stage (56, 60, 63)	Relatively easy to administer and requires few specialty skills (65)		
			An ideal choice for targeting the organs in the abdominal cavity (66)		
Disadvantages	Significant challenges in accessing deep lesions (54, 55, 58, 59)	Requires highly selective tissue targets (55)	Absorbed slower than intravenous injection (65)	Limited to central nervous system tumors (68)	Applied only to small animals in which veins are difficult to find (65)
	Complex procedures make repeat dosing difficult (61, 63)	Physiological barriers such as blood-brain barrier and oncolytic virus elimination by the immune system (55)			
	Mostly applied *in vitro* experiments (39, 42, 44–47)	This route of administration, if any, would be the most likely to lead to toxicity (65)			

In some *in vivo* studies of tumors, researchers have difficulty injecting oncolytic an virus directly into tumors, such as astrocytoma; therefore, intravenous administration is a favorable choice (54, 55, 58, 59). Additionally, intravenous administration of an oncolytic virus has the advantages of convenience and rapidity, which are more suitable at the clinical trial stage (56, 60, 63). However, intravenous administration of oncolytic viruses requires highly selective target tissues (55). That is to say, this route of administration, if any, would be the most likely to lead to toxicity (65). Thus, blindly increasing the application concentration of the virus to compensate for its lack of selectivity will inevitably increase the public concern about the safety of oncolytic virotherapy.

Additionally, those considering use of intravenous administration of an oncolytic virus also need to consider the existence of physiological barriers, such as blood-brain barrier, and the elimination of oncolytic virus by the immune system (55). Is it possible that a wide variety of oncolytic viruses can bypass the blood-brain barrier? In what proportion can the viruses penetrate the blood-brain barrier? How can the immune system of the host be prevented from completely eliminating the injected oncolytic viruses?

The remaining three delivery routes are used less frequently and are mainly limited to animal experiments mostly because of their low efficiency and narrow range of effectiveness. Intraperitoneal injected drugs are absorbed slower than drugs delivered by intravenous injection, although it is an ideal choice for targeting the organs in the abdominal cavity. And subcutaneous injection is applied only to small animals whose veins are difficult to find. Similarly, the scope of intrathecal injection is limited to central nervous system tumors.

In summary, the current choice of the routes for delivery of oncolytic viruses is mainly based on the research purpose and material. No clear criteria or guidelines for choosing between the intratumor and intravenous administration of oncolytic virus have been established, and these approaches need to be further explored by researchers to provide more conclusive evidence for establishing selection criteria.

## Biosafety of Oncolytic Virotherapy

### Adverse Events Induced by Oncolytic Viruses

Oncolytic virotherapy was first used in a clinical trial of cervical cancer in 1956 (70). Since then, in pace with the success of tumor immunotherapy, scientists have paid more attention to oncolytic virotherapy. There is growing recognition that oncolytic virotherapy has the potential to be a safe treatment for cancer patients.

This review consulted 104 clinical trials of oncolytic virotherapy in the PubMed database. After summarizing the results of these 104 clinical trials, we found that the most common adverse events associated with oncolytic virotherapy were mild flulike symptoms and local reactions at the injection sites. Flulike symptoms caused by oncolytic virotherapy often manifested as fever, chills, myalgia, fatigue, nausea, diarrhea, vomiting, headache, etc. (63, 71–73), primarily a grade I–II flulike syndrome. Few patients experienced grade III-IV flulike syndrome (71, 74, 75). Some flulike symptoms disappeared spontaneously during the treatment process, and patients responded well to non-steroidal anti-inflammatory drugs. In addition, a predose of acetaminophen before initiation of the oncolytic virotherapy could reduce these symptoms (76). Local reactions often manifested as pain, rash, erythema, peripheral edema, etc. (77, 78), which spontaneously disappear a few days later or after symptom treatment, and most of the treatments did not induce dose-limiting toxicity (79). Moreover, some common adverse events, including anemia, leukopenia, lymphopenia, neutropenia, thrombocytopenia, liver dysfunction, and hematological abnormalities, specifically emerged in the trials of reovirus, HSV, and adenovirus (80–83). Some patients experienced liver dysfunction as a result of the liver and spleen tropism of the adenovirus (84–86).

Few oncolytic virotherapies cause severe adverse events that harm patients' health, and those that were induced could be managed, rarely causing severe damage to patients. However, oncolytic HSV caused severe hypotension, tachycardia, pleural effusion, herpes virus infection, central nervous system symptoms (such as brain edema, speech disorder, encephalitis, seizures, etc.) in clinical trials (44, 77, 87–89). In addition, oncolytic adenovirus caused pleural effusion, dehydration, hypokalemia, severe liver dysfunction, and sepsis in clinical trials (90–92). Severe hematological abnormalities (leukopenia, lymphopenia, and neutropenia), hypokalemia and pancreatitis were observed in the trials of oncolytic pox virus (93–96). All of the abovementioned virus treatments posed a health-threatening risk to patients who participated in these clinical trials. Pleural effusion could lead to dyspnea and even asphyxia. Fortunately, most of these severe adverse events were managed after withdrawal of the treatment or symptomatic treatment, rarely threatening patients' lives (93, 94, 97–99). Moreover, some preventive measures adopted before oncolytic virotherapy prevented patients from experiencing severe adverse events, as proved by the successful prevention of hypotension by patients drinking high volumes of water or being infused with saline (93, 94). The reasons for some severe events could be attributed to the primary diseases of the patients or to tumor progression (50, 100, 101).

Few oncolytic virotherapies caused virus infection symptoms in trials. Oncolytic HSV led to herpes in patients, and pustules were observed in trials of pox virus (44, 102–104). However, the herpes caused by HSV-based treatments could be managed by acyclovir or ganciclovir, which indicated that specific infection symptoms could be treated by antiviral drugs (78, 83).

The common adverse events of oncolytic virotherapies are summarized in Table 3.

**Table 3:** Common adverse reactions to oncolytic virotherapies.

**Virus type**	**Oncolytic virus**	**Engineering for specificity**	**Clinical Trials.gov identifier**	**Indication**	**Common adverse events**	**Severe adverse events**	**References**
HSV	T-VEC	ICP34.5 deletion; US11 deletion; Human GM-CSF insertion	NCT02288897 NCT01740297 NCT01368276 NCT00289016	Melanoma	Grade I-II flulike symptoms (fever, fatigue, nausea, vomiting), local injection reaction (inflammation, erythema and rash at the injection site)	Severe hypotension, tachycardia, cellulitis, dyspnea, pleural effusion	(77)
	HSV1716	ICP34.5 deletion	NCT02031965 NCT01721018	High grade glioma, malignant pleural mesothelioma	Flulike symptoms, headache, back pain	Urinary tract infection, hydrocephalus, seizures, varicella zoster infection	(104)
	G207	ICP34.5 deletion; UL39 disruption	NCT03911388 NCT02457845 NCT00028158	Cerebellar tumor, supratentorial brain tumor	Flulike symptoms, anemia, leukopenia	Brain edema, speech disorder, encephalitis, hepatitis, viral infection	(105, 106)
	HF10	Spontaneously attenuated HSV-1 mutant	NCT02428036 NCT03259425 NCT03252808 NCT03153085	Melanoma, squamous cell carcinoma of the skin, pancreatic cancer	Flulike symptoms, neutropenia, liver dysfunction	Perforated peritonitis, severe liver dysfunction	(107, 108)
	OH2	ICP34.5 deletion; ICP47 deletion; hGM-CSF insertion	NCT03866525	Intestinal cancer			
Adenovirus	VCN-01	E1A deletion; E2F1 insertion; Replace KKTK with RGDK	NCT02045602 NCT02045589	Solid tumor, pancreatic cancer	Weight loss, elevated liver enzymes, thrombocytopenia (rat)	Viremia (rat)	(74)
	CG0070	E3 deletion; GM-CSF insertion	NCT02365818	Bladder Cancer	Bladder spasm, hematuria, dysuria, urgency, flulike symptoms	Severe dysuria, hypotension	(75)
	Ad5-Δ24-RGD	RGD, Delta-24	NCT0056203 NCT01582516	Breast cancer, glioblastoma	Grade I-II flulike symptoms, abdominal pain, anemia, glucose abnormalities	Pleural effusion, dehydration, intestinal obstruction, hypokalemia	(91)
	H101	E1B deletion; Duplication only in p-53 deficient cancer cells			Flulike symptoms, fever, injection site pain, leukopenia, liver dysfunction, hair loss	Severe leukopenia, severe liver dysfunction	(82, 83, 86, 109)
	Onyx-015	E1B deletion			Fever, elevated liver enzymes	Dehydration, hypotension, sepsis	(85)
	CGTG-102	SSTR, TK, RGD, Ad5/3, GM-CSE, Delta-24	NCT01437280 NCT01598129	Solid tumor	Grade I-II flulike symptoms, mild electrolyte disturbances, elevated liver enzymes, anemia	Dyspnea, pulmonary embolism	(84, 101)
	LOAd703	Trimerized CD40L and 4-1BBL (110)	NCT02705196	Pancreatic cancer			
	ICOVIR-5	DM-1 insertion; E2F1 insertion; Kozak insertion; E1A-Δ24 deletion; RGD deletion (111)	NCT01864759	Melanoma	Flulike symptoms, elevated liver enzymes, thrombocytopenia	Severe transaminase elevation, edema	(92)
Pox virus	JX-594	TK deletion; GM-CSF insertion	NCT03206073 NCT02562755 NCT01394939 NCT01387555 NCT00429312	Colorectal cancer, hepatocellular carcinoma, melanoma	Flulike symptoms, hypotension, tachycardia, hypertension, anorexia, myalgia	Pustules, severe leukopenia, severe lymphopenia, severe fever, hypokalemia, severe headache	(93, 94)
	vvDD	VGF deletion;TK deletion					
	GL-ONC1	Attenuated poxvirus, lister strain	NCT02759588 NCT01584284 NCT00794131	Ovarian cancer, head and neck cancer, solid tumor	Flulike symptoms, anorexia, back pain	Pancreatitis	(96)
Reovirus	Reolysin	Wild type	NCT00651157 NCT01533194 NCT01240538 NCT00602277 NCT00503295	Melanoma, multiple myeloma, solid tumor in children, ovarian epithelial cancer, peritoneal cancer	Flulike symptoms, neutropenia, diarrhea	Severe neutropenia, severe diarrhea, elevated liver enzymes, dehydration	(60)
Coxsackie virus	Cavatak	None	NCT00832559 NCT00438009	Head and neck cancer, melanoma			
Measles virus	MV-CEA	CEA	NCT00390299 NCT00408590	Ovarian epithelial cancer, primary peritoneal cancer, fallopian tube cancer	Flulike symptoms, abdominal pain, anorexia	Arthralgia	(78)
	MV-NIS	NIS	NCT02192775 NCT03456908 NCT02919449 NCT03171493 NCT01503177	Multiple myeloma, non-small cell lung cancer, urothelial carcinoma, malignant pleural mesothelioma	Grade I-II flulike symptoms, leukopenia, diarrhea, neutropenia	Neutropenia, leukopenia, anemia	(112)
Newcastle disease virus	NVD	None	NCT01174537	Glioblastoma, sarcoma, neuroblastoma			
Parvovirus	H-1PV	None	NCT01301430	Multiforme glioblastoma		Biliary duct proliferation, hydrocephalus, decreased consciousness	(113, 114)

### Potential Biosafety Issues of Oncolytic Virotherapies

Although current clinical trials of oncolytic virotherapies have led to severe uncontrollable adverse events, further oncolytic virotherapies needs to be promoted with caution. T-VEC, the first oncolytic virus approved by the FDA after a phase III clinical trial, has been used as a novel cancer therapy modality for only 4 years. Moreover, the potential safety issues and long-term adverse events of oncolytic virotherapies remain unclear.

Oncolytic virotherapies can cause latent infections and more severe potential safety problems that may manifest as long-term adverse events in the future. T-VEC is an oncolytic viral drug developed from HSV-1 that can be latent in nerves and thus induce latent infection. Corrigan et al. indicated that the DNA of oncolytic HSV may persistently remain in neuron bodies surrounding the injection site and may induce severe neurological HSV infection from a long-term perspective (88, 97).

Shedding and transmission of oncolytic viruses during therapy have also caused potential safety issues. Currently, T-VECs kill tumor cells through intratumoral delivery and are mainly used for melanoma treatment. However, during T-VEC therapy, the virus may transfer to other body parts of patients or to people in close contact with patients (87, 115). In clinical trials of Ad5-Δ24-RGD, Kimball et al. found frequent virus shedding in patients who received high doses of oncolytic viruses, which could be detected in serum, urine and saliva, and most commonly in saliva, and the shedding proportion seems to have been interrelated with the dose of the oncolytic virotherapy (90). However, intra-arterial hepatic injection did not result in detectable environmental shedding (116). The infectious shedding virus can be transferred throughout patients' own body and to people who are most likely to be exposed to these patients' body fluids, especially patients' family members and health care providers. Viral shedding was detected for HSV, adenovirus, poxvirus, and reovirus treatments, while it was rarely observed in treatments administered with intravenous VSV or poliovirus (117). However, there was no shedding virus detected in body fluids away from the injection site in HSV1617, H-1PV, or REO-10 therapy (44, 80, 118). In addition to infection, viral shedding can cause homologous recombination between an oncolytic virus and a residual-wild type virus. There is a high risk of homologous recombination when two similar viruses infect the same cell, which could produce a pathogenic transgenic virus. This mechanism has not been observed in the administration of oncolytic virotherapies but has been found during vaccine manufacture and usage (119). Still, shedding viruses observed in past studies are quite limited and highly attenuated, which is hard to cause damage to cancer patients, and the dose of oncolytic viruses for cancer patients is too small to cause shedding. In order to minimize the environmental viral shedding, the exposure of healthcare providers should be controlled when administrating oncolytic viruses (119).

Studies on the safety of oncolytic virotherapies for specific populations are currently insufficient. The T-VEC guidelines clearly indicate that people with low immunity or pregnancy should avoid using T-VECs. Wild-type HSV-1 that infects pregnant may cross the placental barrier and influence the fetus (71, 87). Preclinical studies of H-1PV showed that H-1PV induces embryonic and fetal toxicity in rodents and harmful effects on progeny, usually leading to the death of a fetus infected during the second trimester. When pregnant women are infected in the third trimester or a few days before birth, the progeny often develop “osteolytic syndrome” characterized by dwarfism and various down syndrome-like features (120, 121). Cancer patients who received radiotherapy and chemotherapy usually show low immunity to virus infection. Whether it is safe for these patients to be administered oncolytic virotherapy is debated (73). Children with severe combined immunodeficiency (SCID) who received an oncolytic retrovirus were found to have viral genes integrated into the LMO2 proto-oncogene region, which triggered the development of leukemia, reducing the survival rate of these patients with this compromised condition (122).

### Methods to Improve the Biosafety of Oncolytic Virotherapy

To improve the biosafety of oncolytic virotherapies, the following three aspects may be considered. One approach involves selecting viruses that are not infectious to normal tissues. The natural host of parvovirus is rat, so parvovirus is non-pathogenic to humans because of anticellulosic selectivity, resulting in low selectivity for non-malignant tumor cells in humans. The overexpression of cytokines and transcription factors in tumors can active metabolic pathway which regulate function of Non-structural protein 1 (NS1, an essential protein for viral DNA replication, gene expression, and virus-induced cytotoxic effects), which can increase the tumor selectivity of parvoviruses (114, 118). In addition, reovirus has no or low pathogenicity in humans, and its pathogenicity in normal cells can be attenuated by repeated subculturing (80, 119). Kaid et al. reported that ZIKV^BR^ can kill central nervous system (CNS) tumor cells specifically and effectively without causing damage to normal cells and other kinds of tumor cells, which means ZIKV^BR^ have possibility to treat CNS embryonal tumors, and ZIKV^BR^ caused very few infective cases of infants and adults in clinical trials (123).

The second approach involves attenuating the pathogenicity of oncolytic viruses to normal cells by genetic modification of the viruses, many of which have been used in studies. To develop oncolytic HSV-1 drugs, such as T-VEC and G207, the ICP-34.5 gene in HSV1716, which is a neurovirulence factor of HSV, was deleted, attenuating the infectivity of HSV in normal neurons (73, 117, 122, 124, 125). Mutation and deletion of the E1 gene can reduce adenovirus selectivity of normal cells, and this genetic modification was made to the Onyx-015 and H10 viruses (90, 126). Moreover, through genetic manipulation, the arg-gly-asp (RGD) sequence was integrated into the HI loop of the adenovirus capsid protein to enhance the infectivity of the oncolytic adenovirus in tumor cells (90, 127). It was reported that the enhanced liver infection by adenovirus may be caused by the binding between coagulation factor (F) X and hypervariable regions (HVR). Thus, inserting mutations in the FX-binding domain of the HVR and replacing them with HVRs of other serotypes of the original adenovirus can significantly reduce the liver tropism of the oncolytic adenovirus (128). Deleting genes such as TK, VGF, hemagglutinin, and B18R in the oncolytic pox virus can notably reduce its virulence in normal cells (81, 95, 103).

The third approach involves the recombination of different kinds of oncolytic viruses for therapy. The recombination of vesicular stomatitis virus (VSV) and Newcastle disease virus (NDV), named recombinant VSV-NDV (rVSV-NDV), greatly reduced cytotoxicity in healthy hepatocytes and neurons and was not pathogenic to the embryonated eggs. In the rVSV-NDV, the backbone of the VSV is retained. However, its glycoprotein is replaced by hemagglutinin-neuraminidase (HN) and the envelope proteins of the NVD. The adverse events caused by the off-target effects in brain and liver, which were observed in the trials of wild-type VSV, were significantly decreased because of the replacement of the glycoprotein (129). Adenoviruses are widely used in recombinant oncolytic virotherapies, such as those based on adenovirus-coxsackie virus and adenovirus-parvovirus (130). Recombinant adenoviruses and parvoviruses retain the infectivity of the adenovirus and the harmlessness of the parvovirus in normal cells, thereby killing the tumor cells and exempting the normal cells (131).

After analysis of the clinical trials of different types of oncolytic virotherapies, we concluded that oncolytic virotherapies are generally safe, and have a low incidence of adverse events, and cause only slight damage, which can be controlled or spontaneously regress (132). Clinical trials of oncolytic viruses such as G207, HSV1716, NV1020, Ad [I/PPT-E1A] and reovirus have proven their biosafety and effectiveness in tumor therapy (44, 73, 80, 106, 125). The HSV-2 and measles viruses were also proven non-toxic to humans in a number of mammalian experiments (124, 133–135). However, oncolytic virotherapies have unpredictability problems, such as long-term adverse events, which still need to be closely observed. As clinical trials expand and more patients participate, more long-term or short-term adverse events will likely be reported and analyzed. In addition, with new discoveries of oncolytic virotherapies, increasing numbers of genetic modifications to oncolytic viruses, and additional recombinant viruses, microRNAs, and viral vectors found, the safety of oncolytic viruses in tumor immunotherapy will be further guaranteed (117, 136, 137).

## Conclusion and Perspective

In the past decade, oncolytic virotherapy, as a tumor immunotherapy, has emerged as a promising approach because of its selective killing of tumors (summarized in Supplementary Table 1). Currently, many clinical trials are ongoing, and most studies focus on how to improve the efficiency of oncolytic virotherapy, for which genetic modification, combination therapy and increasing viral replication and spreading are of specific interest. Moreover, the application of oncolytic viruses to tumor imaging is also under investigation.

This review summarizes the pros and cons of various routes of virus delivery and the biosafety of oncolytic virotherapy. Currently, oncolytic viruses are primarily administered through intratumoral and intravenous delivery, with each having advantages and disadvantages. The specific choice of which route of delivery is made without a clear standard or criterion and is mainly selected to reduce adverse events and enhance efficacy. Because of the advancement of virus recombination and genetic modification, as well as the specific mechanisms of oncolytic virotherapy, severe adverse events caused by oncolytic virotherapy have rarely been reported, while milder adverse events can generally be controlled or disappear spontaneously. Therefore, oncolytic virotherapy is currently generally safe, potential safety issues that are not currently presented or detected cannot be eliminated.

For virus delivery, first, more attention should be paid to maximizing the effective viral load in tumor lesions, thereby improving oncolytic virotherapy efficacy, which is also based on improved tumor selectivity. Second, similar to the that of drug combination therapy, different routes of delivery can be combined to enhance oncolytic virotherapy efficacy. In recent years, relevant clinical trials have been carried out (NCT01301430). However, the best application of oncolytic virotherapy is related to personalized medicine; that is, specific viral delivery to specific tumors forms a one-to-one precision therapeutic mode and may be the direction of oncolytic virotherapy development.

With regard to biosafety issues, a great majority of oncolytic virotherapy remains in phase II/III clinical trials. There is still a long road ahead before virotherapy is widely applied in tumor therapy. Although oncolytic virotherapy rarely caused fatal adverse events in recent clinical trials, continued studies should actively seek a balance between enhancing efficacy and reducing adverse events through virus modification or other means, with the goal of minimizing the adverse events as much as possible on the basis of ensuring treatment efficacy, which would also show the absolute advantage of oncolytic virotherapy over traditional tumor therapy such as surgical resection and adjuvant chemoradiation. Future studies should increasingly focus on the specific mechanism of the interaction between oncolytic viruses and the human immune system and the tumor immune microenvironment, thus preventing adverse events from the source of these viruses and latent virus infection and virus shedding and transmission, which would result in overall improved patient safety. In addition, more attention should be paid to the tumor selectivity of specific oncolytic virotherapies. By developing new viral vectors targeting tumor cells, oncolytic viruses can attach to tumor lesions with affinity but not to normal tissues, thereby eliminating adverse events and ensuring safety.

The need to choose the delivery mode and ensure the biosafety of the oncolytic virotherapy provides an important impetus to transform existing research results into clinical translations and plays a decisive role in guiding future clinical applications. It is expected that oncolytic virotherapy will become a powerful weapon for the therapy of malignant tumors. In the future, if oncolytic virotherapy can be developed into an oral preparation instead of an injection, delivered through a novel vector, specified by genetic modification or recombination, and effectively reach tumor lesions or reach an effective concentration through intestinal absorption, such that it exerts resistance against cancer cells and does not harm to normal tissues, then oncolytic virotherapy will be a revolutionary development in the new generation of tumor immunotherapy.

## Author Contributions

LL, SL, DH, BT, and JM analyzed the literatures and studies and wrote the manuscript.

### Conflict of Interest

The authors declare that the research was conducted in the absence of any commercial or financial relationships that could be construed as a potential conflict of interest.
