# Supplementary material for: Delivery and Biosafety of Oncolytic Virotherapy
Source: Front Oncol. 2020 Apr 16;10:475. doi: 10.3389/fonc.2020.00475 (PMC7176816; doi:10.3389/fonc.2020.00475)
Supplement: Supplementary file 1 [file Table_1.pdf]

Supplementary Table 1. Delivery routes and the biosafety of oncolytic virus

	Virus type	Oncolytic virus	Indication	Reference
**Delivery routes**				
Intratumoral administration	HSV	HSV1716	Extracranial cancers	(44)
	Adenovirus	VCN-01	Retinoblastoma	(46)
	Vesicular stomatitis virus	VSVΔ51	Melanoma	(39)
	Alphavirus	M1	Glioblastoma	(40)
	HSV	HF10	Pancreatic carcinoma	(50)
	Reovirus	Reovirus	Astrocytoma	(51)
	Reovirus	Reovirus	Gliomas	(48)
	Poxvirus	CF33-hNIS	Colon cancer	(45)
	Poxvirus	CF33	Triple-negative breast cancer	(47)
	Poxvirus	CF33-Fluc	Colorectal cancer	(69)
Intravenous administration	Reovirus	Reovirus	Astrocytoma	(53)
	Vaccinia virus	OVV-CXCR4-A-Fc	Neuroblastoma	(54)
	HSV	G207	Supratentorial malignant tumors	(56)
	Poxvirus	Poxvirus	Neuroblastoma	(55)
	Adenovirus	Ad-IAI.3B	Lung squamous cell carcinoma	(57)
	Alphavirus	M1	Bladder cancer	(58)
	Reovirus	Reovirus	Carcinoma ovarii, Carcinoma of fallopian tube, Peritoneal carcinoma	(60)
	Maraba virus	MG1	Prostatic carcinoma	(59)
	Adenovirus	Ad6	Prostate cancer	(61)
	HSV	T-VEC	Melanoma	(63)
	Measles virus	MV-GFP	Atypical teratogenic rhabdoid tumor	(64)
Intraperitoneal administration	HSV	oHSV-1	Kaposi's sarcoma, angiosarcoma, epithelioid sarcoma	(65)
	Adenovirus	ONCOS-102	Gastrointestinal stromal tumor, leiomyosarcoma, liposarcoma	(67)
	Poxvirus	CF33	Pancreatic carcinoma	(66)
Intrathecal treatment	Poliovirus	PVS-RIPO	Glioblastoma, Glioma, Ependymoma, Primitive neuroectodermal tumor,	(68)

			Central nervous system lymphoma	
Subcutaneous administration	Adenovirus	ONCOS-102	Melanoma, soft tissue sarcoma	(67)
**Biosafety**				
Common adverse events (only)				
Severe adverse events (only)	Parvovirus	H-1PV	Multiforme glioblastoma	(113, 114)
Both Common and Severe adverse events	HSV	T-VEC	Melanoma	(77)
	HSV	HSV1716	High grade glioma, malignant pleural mesothelioma	(104)
	HSV	G207	Cerebellar tumor, supratentorial brain tumor	(105, 106)
	HSV	HF10	Melanoma, squamous cell carcinoma of the skin, pancreatic cancer	(107, 108)
	Adenovirus	VCN-01	Solid tumor, pancreatic cancer	(74)
	Adenovirus	CG0070	Bladder Cancer	(75)
	Adenovirus	Ad5-Δ24-RGD	Breast cancer, glioblastoma	(91)
	Adenovirus	H101		(82, 83, 86, 109)
	Adenovirus	Onyx-015		(85)
	Adenovirus	CGTG-102	Solid tumor	(84, 101)
	Adenovirus	ICOVIR-5	Melanoma	(92)
	Pox virus	JX-594	Colorectal cancer, hepatocellular carcinoma, melanoma	(93, 94)
	Pox virus	GL-ONC1	Ovarian cancer, head and neck cancer, solid tumor	(96)
	Reovirus	Reolysin	Melanoma, multiple myeloma, solid tumor in children, ovarian epithelial cancer, peritoneal cancer	(60)
	Measles virus	MV-CEA	Ovarian epithelial cancer, primary peritoneal cancer, fallopian tube cancer	(78)
	Measles virus	MV-NIS	Multiple myeloma, non-small cell lung cancer, urothelial carcinoma, malignant pleural	(112)

			mesothelioma
Neither Common or Severe adverse events (No reports yet)	HSV	OH2	Intestinal cancer
	Adenovirus	LOAd703	Pancreatic cancer
	Pox virus	vvDD	
	Coxsackie virus	Cavatak	Head and neck cancer, melanoma
	Newcastle disease virus	NVD	Glioblastoma, sarcoma, neuroblastoma

Note:

Common adverse events include flulike symptoms (fever, fatigue, nausea, vomiting), local injection reaction (inflammation, erythema and rash at the injection site), headache, back pain, anemia, leukopenia, neutropenia, liver dysfunction, weight loss, elevated liver enzymes, thrombocytopenia , bladder spasm, hematuria, dysuria, urgency, abdominal pain, glucose abnormalities, hair loss, mild electrolyte disturbances, hypotension, tachycardia, hypertension, anorexia, myalgia, diarrhea.

Severe adverse events include severe hypotension, tachycardia, cellulitis, dyspnea, pleural effusion, urinary tract infection, hydrocephalus, seizures, varicella zoster infection, brain edema, speech disorder, encephalitis, hepatitis, viral infection, perforated peritonitis, severe liver dysfunction, viremia, severe dysuria, dehydration, intestinal obstruction, hypokalemia, severe leukopenia, sepsis, pulmonary embolism, edema, pustules, severe lymphopenia, severe fever, severe headache, pancreatitis, severe neutropenia, severe diarrhea, arthralgia, biliary duct proliferation, decreased consciousness.

The references of the right column are listed in the main text of this publication.
